# Amphotericin B, an Anti-Fungal Medication, Directly Increases the Cytotoxicity of NK Cells

**DOI:** 10.3390/ijms18061262

**Published:** 2017-06-13

**Authors:** Nayoung Kim, Ji-Wan Choi, Hye-Ran Park, Inki Kim, Hun Sik Kim

**Affiliations:** 1 Department of Convergence Medicine, Asan Medical Center, University of Ulsan College of Medicine, Seoul 05505, Korea; naykim@amc.seoul.kr (N.K.); ik.kim@amc.seoul.kr (I.K.); 2 Asan Institute for Life Sciences, Asan Medical Center, University of Ulsan College of Medicine, Seoul 05505, Korea; cn1vet202@naver.com; 3 Department of Biomedical Sciences, University of Ulsan College of Medicine, Asan Medical Center, Seoul 05505, Korea; buforin@naver.com; 4 Department of Microbiology, University of Ulsan College of Medicine, Asan Medical Center, Seoul 05505, Korea

**Keywords:** immunomodulatory drugs, library screening, amphotericin B, natural killer cells, cytotoxicity

## Abstract

Immunomodulatory drugs (IMiDs) present one example of immunomodulatory agents that improve cancer immunotherapy. Based on the cytotoxic activity of natural killer (NK) cells against cancer cells, a high throughput screening method for the identification of novel immunomodulatory molecules with the potential to stimulate NK cell cytotoxicity against cancer cells was designed and tested using an approved drug library. Among the primary hit compounds, the anti-fungal drug amphotericin B (AMP-B) increased the cytotoxicity of NK cell line and human primary NK cells in a direct manner. The increase in NK cell activity was related to increased formation of NK-target cell conjugates and the subsequent granule polarization toward target cells. The results of the present study indicate that AMP-B could serve a dual function as an anti-fungal and immunomodulatory drug.

## 1. Introduction

Cancer immunotherapy aims to stimulate the immune system to treat cancer and has shown promise in preclinical and clinical settings. However, the results are inconsistent between cancer patients and unsatisfactory in certain types of cancer. A variety of new therapeutic agents have become available to improve the efficacy of cancer immunotherapy [1,2]. Among others, monoclonal antibodies (mAb) that block immune checkpoints (e.g., PD-1, CTLA-4) or trigger T cell co-stimulation (e.g., OX-40, 4-1BB) are well-known examples of the immunomodulatory agents. Another promising class is represented by immunomodulatory drugs (IMiDs) that potentiate activities of immunotherapy alone or in combination with therapeutic Ab and adoptive cell transfer.

The search for IMiDs for cancer immunotherapy has been fruitful to a certain extent. Thalidomide and its derivatives, such as lenalidomide, are a classic example of drug repositioning/repurposing for cancer therapy. Originally developed as sedatives, these agents are currently used to treat multiple myeloma [3]. They have anti-cancer, anti-angiogenic, and immune-stimulating effects, in particular on natural killer (NK) cells [4]. However, the effects of these drugs on NK cells may be indirectly mediated by cytokines, such as interferon (IFN)-γ and interleukin (IL)-2 from stimulated T cells and dendritic cells (DCs) [5,6]. Other drugs such as bortezomib also modulate NK cell activity, although the mechanisms of action are mostly indirect [4]. These results imply that these agents may not be effective in isolated or in vitro expanded NK cells for adoptive therapy. In this respect, there remain unmet needs for novel IMiDs that directly stimulate NK cells.

NK cells play a quintessential role in anti-cancer and anti-viral immunity by killing target cells directly and by producing chemokines and cytokines, such as macrophage inducible protein (MIP)-1α/β and IFN-γ. The cytotoxicity of NK cells involves several steps: activation, mediated by engaging activating receptors and releasing inhibitory receptors; cell–cell adhesion; granule polarization; and degranulation [7]. NK cells express multiple activating and inhibitory receptors with different functions. For instance, the engagement of CD16 induces degranulation without polarization, whereas that of lymphocyte function-associated antigen (LFA)-1 induces granule polarization, but not degranulation [8]. Degranulation is mediated by the Vav1-dependent activation of phospholipase Cγ and subsequent calcium fluxes [9,10]. The granules, which contain cytolytic molecules such as perforin and granzyme, are responsible for eliminating cancer and virus-infected cells. Unlike T cells, NK cells can remove cancer cells rapidly without major histocompatibility complex (MHC) restriction, and immune cell therapy using NK cells was therefore developed for cancer treatment [11]. The identification of effective and safe methods to stimulate NK cells in vitro and in vivo would be desirable.

The development of high throughput screening (HTS) systems has facilitated the discovery of novel therapeutics as well as drug repositioning [12]. The whole process of HTS is performed on microtiter plates to select compounds with inhibition or activation effects, which is called hit selection. The grading methods to select hits include statistical standard scores, such as *Z*-scores. Without fully automatic HTS, drug discovery would have been painstaking procedures going through dozens of thousands of chemicals. Approved drug libraries are useful resources, as they comprise in-use or previously approved drugs that are in general well characterized and can safely be administered (e.g., Prestwick Chemical Library). In more than 190 publications citing the library, the minimal sequence of the regulator of calcineurin (RCAN), a vasodilator, is suggested as an immune suppressant that inhibits the calcineurin-nuclear factor of activated T cells pathway [13]. A recent study showed that many molecules, including two ergosterol synthesis inhibitors, modulate Fc-mediated human NK cell activation [14]. Here, we designed a HTS system to identify novel immunomodulatory molecules from Prestwick Chemical Library with the potential to stimulate NK cell natural cytotoxicity against cancer cells. We showed that amphotericin B (AMP-B), an anti-fungal agent, increased the cytotoxicity of NK cells by promoting conjugate formation and granule polarization.

## 2. Results

### 2.1. Amphotericin B (AMP-B) Increased the Cytotoxicity of a Human NK Cell Line

**Figure 1:**
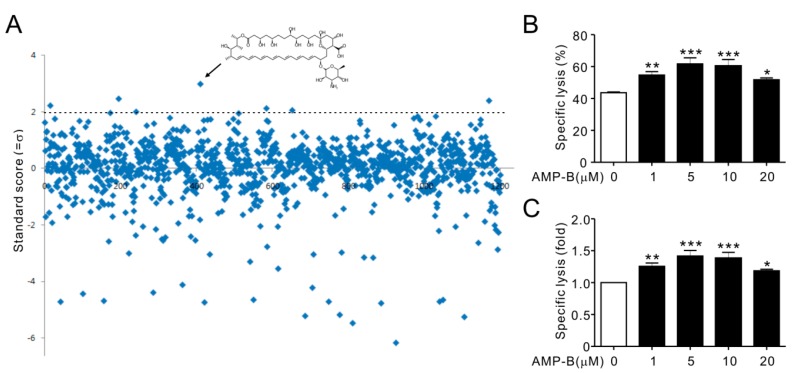
Amphotericin B (AMP-B) increased natural killer (NK) cell cytotoxicity. NKL cells were seeded into 96-well plates and pretreated with compounds (5 µM) from the Prestwick Chemical Library at 37 °C for 1 h. NKL cells were then incubated with 221 target cells (effector (E):target (T) ratio 5:1) for 2 h. The cytotoxicity of NKL cells was assessed using the Europium assay. (**A**) Normalized standard score distribution for the 1200 small molecule screen. The +2σ cut-off value was used for hit definition and indicated by a dotted line. The arrow indicates AMP-B; (**B**) Lysis (%) of 221 cells by NKL cells (10:1 E:T ratio) pretreated with the indicated concentrations of AMP-B; (**C**) The relative lysis of 221 cells by AMP-B-treated NKL cells is expressed as fold change. Data represent the mean ± SD of three independent experiments. * *p* < 0.05, ** *p* < 0.01, and *** *p* < 0.001.

To identify immunomodulatory small molecules for human NK cells, a high throughput screening system was established to measure cytotoxicity using the Europium assay, and the Prestwick Chemical Library was screened. The activity of each compound was evaluated at 5 µM using NKL cells, a human NK cell line, and 721.221 (hereafter referred to as 221) cells as target cells. The efficacy of each molecule for increasing cytotoxicity was quantified by calculating the Z-score (standard score). Six compounds with a *Z*-score > 2.0 were identified; heptaminol hydrochloride (2.21), asenapine maleate (2.45), AMP-B (2.97), yohimbine hydrochloride (2.11), picrotoxinin (2.04), and tulobuterol (2.38) (Figure 1A). The Z′-factor for the total screen was 0.755, indicating that the screening was performed with high resolution. The six chemicals were subjected to first and second cherry-pick tests, and AMP-B, with a Z-score of 2.97 (Figure 1A, black arrow), induced the highest and consistent increase in cytotoxicity. In comparison, statistical significance of increasing NK cytotoxicity was inconsistent with other five hits (data not shown). As shown in Figure 1B,C, AMP-B increased the cytotoxicity of NKL cells by approximately 25% and 50% at 1 and 5 µM, respectively, which were optimal concentrations for increasing NK cell cytotoxicity. Higher concentrations had a negative effect on cytotoxicity.

### 2.2. AMP-B Increased the Cytotoxicity of Ex Vivo and Expanded Human Primary NK Cells

**Figure 2:**
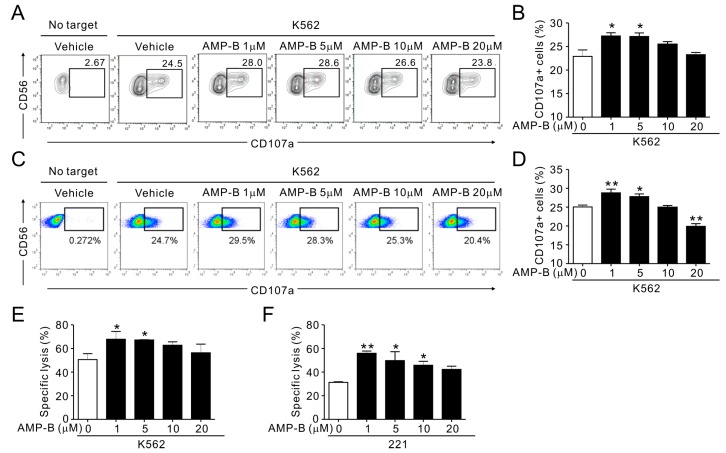
AMP-B increased the natural cytotoxicity of primary NK cells. (**A**,**B**) PBMCs were pretreated for 1 h with the indicated doses of AMP-B and incubated with target cells (K562) for 2 h in the presence of AMP-B. Degranulation of NK cells was measured by cell surface expression of CD107a on CD3-CD56+ NK cells. (**A**) Representative flow cytometry profiles showing the percentages of CD107a+ NK cells; (**B**) Summary graphs of statistical bar charts showing the expression of CD107a by NK cells. Mean values ± SEM of three independent experiments are shown. (**C**,**D**) Primary NK cells after expansion were preincubated for 1 h with the indicated doses of AMP-B and mixed with K562 target cells for 2 h in the presence of AMP-B and fluorochrome-conjugated anti-CD107a monoclonal antibody (mAb). Cells were then stained with fluorochrome-conjugated mAb to CD56, and the level of CD56+CD107a+ NK cells was analyzed by flow cytometry. Shown are representative flow cytometry profiles (**C**) and summary graphs of statistical bar charts (**D**) demonstrating expression of CD107a by NK cells. The mean values ± SD of three independent experiments are shown. (**E**,**F**) Lysis (%) of K562 (**E**) or 221 (**F**) target cells by primary expanded NK cells for 1 h that were pretreated with AMP-B as described in (**C**) (2:1 E:T ratio). The mean values ± SD of three independent experiments are shown. * *p* < 0.05 and ** *p* < 0.01.

The properties of established NK cell lines may differ from those of primary NK cells. Therefore, the potential of AMP-B to stimulate NK cell cytotoxicity was assessed using human primary NK cells. Fresh peripheral blood mononuclear cells (PBMCs) were activated by IL-2 for 24 h and then pretreated with AMP-B for 1 h. NK cell cytotoxicity correlates with the degranulation efficiency of NK cells [8]. As shown in Figure 2A, AMP-B increased degranulation, as indicated by increased CD107a expression in response to K562 target cells. The results are summarized in Figure 2B, which shows that 1–5 µM AMP-B induced the moderate but significant CD107a expression. IL-2- and IL-15-expanded NK cells, which were previously tested in clinical trials for hematological malignancies, showed similar moderate but statistically significant increases in CD107a expression induced by AMP-B at the same concentrations (Figure 2C,D). The results with expanded NK cells were confirmed using the Europium-based cytotoxicity assay in 221 cells (Figure 2F) and K562 cells (Figure 2E). Primary NK cells were more sensitive to AMP-B treatment at higher concentrations than NKL cells, as indicated by a greater decrease in cytotoxicity at 10–20 µM. AMP-B had a stronger effect on increasing the cytotoxicity of primary NK cells at 1 µM than at 5 µM, although the cytotoxicity increase was statistically significant at both 1 and 5 µM. Taken together, these results indicated that AMP-B increased the degranulation and cytotoxicity of ex vivo NK cells and in vitro expanded NK cells.

### 2.3. Amp-B Accelerated Conjugate Formation between NK Cells and Target Cells

**Figure 3:**
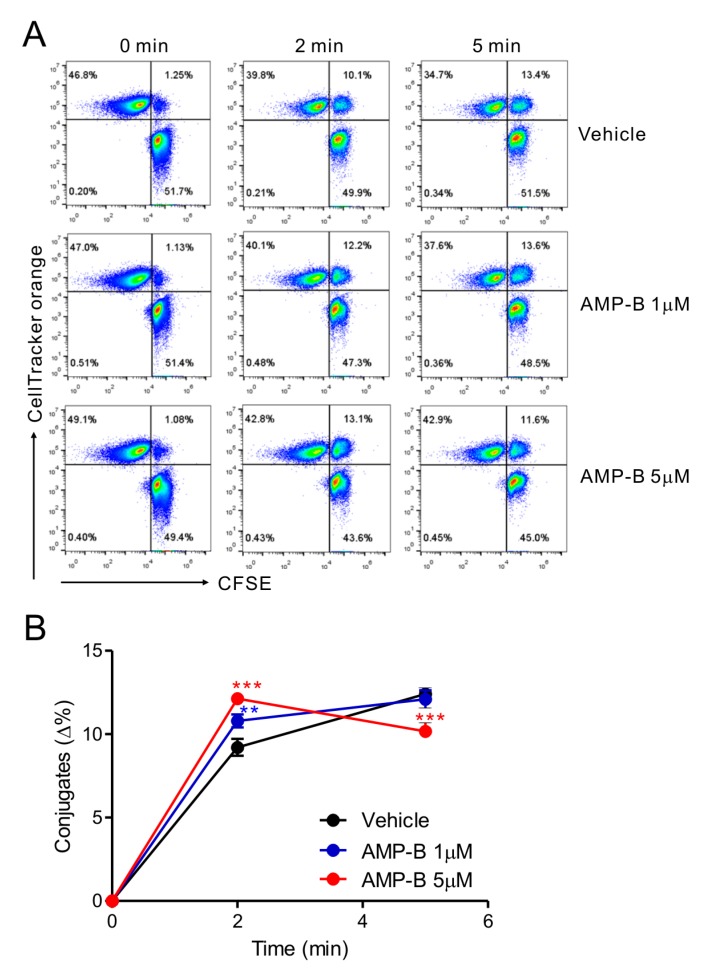
AMP-B increased conjugate formation in NK-target cells. NKL cells loaded with CFSE were treated with the indicated doses of AMP-B for 30 min and then incubated with CellTracker orange CMTMR-labeled 221 target cells at an E: T ratio of 1:1 for the indicated time points. Cells were then fixed and analyzed by flow cytometry to detect conjugate formation, as represented by the double-positive population in the upper right quadrant. Shown are representative flow cytometry profiles of two independent experiments (**A**) and summary graphs of statistical line charts (**B**) showing conjugate formation between NKL cells and 221 cells. ** *p* < 0.01 and *** *p* < 0.001.

To understand the mechanisms of action of AMP-B on NK cells, the sequential steps leading to NK cell cytotoxicity were investigated. Because cytotoxicity could be promoted by increased cell–cell interactions, a cell adhesion assay was performed using NKL cells and 221 target cells. AMP-B promoted the formation of conjugates between NKL and 221 cells in a short time (Figure 3A). A modestly but significantly increased rate of conjugate formation was observed in a dose-dependent manner at 2 min following treatment with 1–5 µM AMP-B; however, increased conjugation was maintained for a short time, with significant dissociation observed at 5 min in response to 5 µM AMP-B (Figure 3B). In comparison, higher concentrations of AMP-B (10–20 µM), which had a negative effect on cytotoxicity, caused a decrease of conjugate formation even at 2 min compared with lower concentrations of AMP-B (1–5 µM). Subsequent lytic synapse formation or cytolytic granule polarization could not be assessed by flow cytometry. Therefore, confocal microscopic analysis was performed for further mechanistic studies.

### 2.4. Granule Polarization Was Enhanced by AMP-B

**Figure 4:**
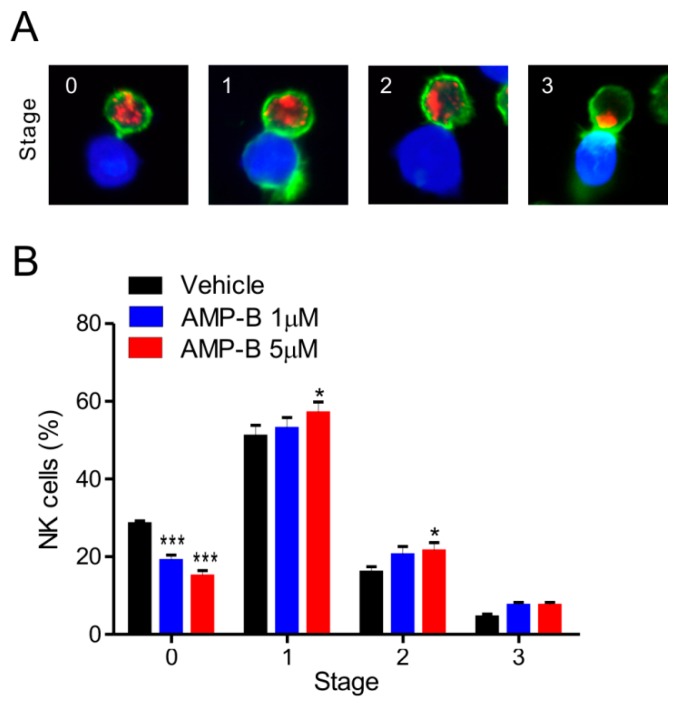
AMP-B increases cytolytic granule polarization toward target cells. (**A**) Representative confocal images of conjugates between NKL cells and CellTracker orange CMTMR-labeled 221 cells (**blue**). Conjugates were fixed, permeabilized, and stained with phalloidin for actin (**green**) and anti-perforin antibody (**red**). Conjugates were then analyzed by confocal microscopy to determine the polarization of perforin-containing granules toward target cells. Conjugates were categorized into different stages according to the progression of granule polarization toward target cells. Shown are conjugates representative of each stage; (**B**) NKL cells pretreated for 30 min with the indicated doses of AMP-B were incubated with CMTMR-loaded 221 cells for 30 min. Cells were then stained as described in (**A**), and the percentages of NKL cells at each stage of granule polarization were measured with at least 100 NKL-target cell conjugates. Summary graphs of statistical line charts are shown. * *p* < 0.05 and *** *p* < 0.001.

**Figure 5:**
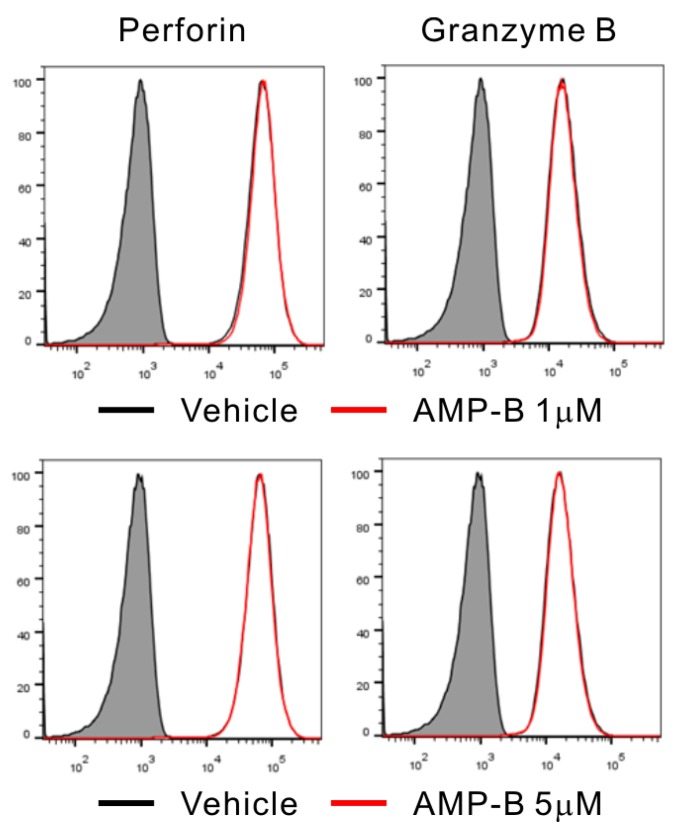
Effect of AMP-B on the expression of perforin and granzyme B in NK cells. Representative flow cytometry profiles showing the expression of the perforin (**left**) and granzyme B (**right**) in NKL cells after incubation with 1 µM (**upper**) or 5 µM (**bottom**) of AMP-B for 3 h (**red solid line**), or with vehicle only (black solid line). Isotype control staining is shown as a shaded histogram. Shown is a representative of two independent experiments.

Polymerized actin accumulating in the lytic synapse and cytolytic granules was detected by phalloidin and anti-perforin staining, respectively. Based on this approach, different conjugate stages were defined as follows: 0, conjugates lacking actin polymerization and granule polarization; 1, conjugates with polymerized actin and without granule polarization; 2, conjugates in which perforin-containing granules partially polarized toward the lytic synapse; and 3, conjugates in which granules fully polarized toward the lytic synapse. Actins (green) are reorganized to form polymerized F-actin in Stage 1, and then granules containing perforin (red) are polarized to the synapses in Stages 2 and 3 (Figure 4A). As shown in Figure 4B, AMP-B treatment increased granule polarization in Stages 1–2 in a dose-dependent manner. Statistical analysis revealed that 5 µM AMP-B caused a significant increase of granule polarization in Stages 1-2 relative to a significant decrease in Stage 0. Similar situation also occurred in response to 1 µM AMP-B, but the statistical significance was observed in Stage 0. The increase induced by AMP-B was also observed in Stage 3. Further co-incubation with AMP-B at 5 µM may increase NK cell numbers in Stage 3, as NK cells increased by 5 µM AMP-B in Stage 1 are expected to reach Stage 3 with additional time. The relative decrease in Stage 0 could reflect such increase in Stages 1–3. The results shown in Figure 3 and Figure 4 indicated that the rapid increase in conjugate formation may result in a gradual induction of granule polarization. However, AMP-B did not cause the increase in calcium flux required for degranulation or the intracellular expression levels of perforin and granzyme B (Figure 5). Collectively, these results suggest that increased conjugate formation and subsequent granule polarization are likely the mechanisms underlying the effect of AMP-B on increasing NK cell cytotoxicity.

## 3. Discussion

In this study, we found that AMP-B increased the natural cytotoxicity of human NK cells by promoting conjugate formation and granule polarization. AMP-B is an anti-fungal agent isolated from *Streptomyces nodosus*. It can be administered orally and intravenously to treat thrush and systemic fungal infections. AMP-B was previously shown to have immunostimulatory effects on human lymphokine-activated killer (LAK) cells [15]. A large proportion of LAK cells are NK cells. AMP-B at 1 mg/L increases the cytotoxicity of human LAK cells against Daudi cells by 50% compared with the effect of vehicle control. A dose of 1 mg/L AMP-B corresponds to approximately 1.08 µM, which is sufficient to increase the cytotoxicity of NK cells according to our current study. AMP-B is also suggested as an adjuvant molecule for murine and bovine γδ T cell responses [16]. In this report, AMP-B injection into mice increased IFN-γ production, which could be produced by NK cells and T cells. Furthermore, γδ T cells share many properties with NK cells, and they play a role in innate immunity through various NK cell receptors such as natural killer group 2D (NKG2D). This suggests that AMP-B is a potential candidate for cancer immunotherapy mediated by NK cell activation.

AMP-B was also shown to have inhibitory effects on murine NK cells [17]. In that study, the repression was significant at relatively high concentrations of AMP-B (5–10 mg/L, approximately 5.4–10.8 µM), which is compatible with our results using human primary NK cells despite some differences in NK cell activation between mice and men [18,19]. Given the stimulation of NK cells by AMP-B at low concentrations [15,20], the effect of AMP-B on NK cell activation appears to be dose-dependent. Furthermore, immunomodulatory effects of AMP-B became manifest upon exposure to cytokines such as IL-2 and IL-18, a situation that likely occurs during the course of infection and transformation. In support, we observed that freshly isolated, rather than IL-2-primed, NK cells did not respond to AMP-B for enhancing cytotoxicity, an observation compatible with no effect of AMP-B on spontaneous NK cell activity [17]. Thus, we anticipate that systematic studies would be required to establish the effect of AMP-B on NK cells in the context of various pathophysiological conditions.

Understanding how existing medications modulate or affect immune systems is important. AMP-B can be administered to transplant patients with fungal infections who are vulnerable to tumors. In this case, AMP-B may have a dual function in treating the fungal infection and enhancing activation of NK cells and γδ T cells. AMP-B can also be added during in vitro expansion of NK cells for adoptive immune cell therapy. However, it is possible that higher concentrations of AMP-B are rather toxic to NK cells, and primary NK cells appear to be more sensitive to AMP-B than immortalized NK cells, as shown here. AMP-B is an anti-eukaryotic agent and could elicit severe side-effects in humans [21]. The mean concentrations of AMP-B in the sera during treatment for systemic mycoses were reported at 1.21, 0.62, and 0.32 µg/mL after 1, 18, and 42 h, respectively [22]. The concentrations in human blood are appropriate for NK cell stimulation and are not inhibitory or toxic according to our current study. Therefore, AMP-B could be used to stimulate NK cells in vivo at safe concentrations; however, the doses should be optimized for clinical trials, considering the biphasic effect of AMP-B on NK cell activation.

Combination therapy with conventional chemotherapeutic agents or immunotherapies needs to be considered to improve the anti-cancer effect. Certain chemotherapeutic agents were recently shown to modulate NK cell function. Azacytidine impairs NK cell reactivity, whereas decitabine at 5 µM increases it toward stimulation [23] despite the fact that azacytidine (5-azacytidine) and decitabine (5-aza-2′-deoxycytidine) are both cytosine analogues. Dasatinib, a BCR-ABL/SRC inhibitor, increases NK cell cytotoxicity and cytokine production [24]. Bortezomib, a proteasome inhibitor used as an anti-cancer drug or immunosuppressant, can sensitize tumor cells to NK cell-mediated lysis [4]. These data indicate that combination therapy with conventional cancer drugs and immunotherapies can be designed to improve patient outcomes. However, comprehensive preclinical and clinical trials are needed, particularly to identify possible drug interactions.

The same chemical library was used to identify butenafine and naftifine, which are ergosterol synthesis inhibitors, as agents capable of increasing CD107a expression in antibody-dependent cellular cytotoxicity (ADCC) [14]. However, these compounds did not show a significant effect in our current study. The concentration of 10 µM used in the previous study did not significantly increase NK cell cytotoxicity in our study. In addition, we analyzed natural cytotoxicity against tumor cells, rather than ADCC mediated by CD16. NK cell cytotoxicity can be activated by the balanced engagement of different and multiple receptors, such as NKG2D, NCRs, 2B4, LFA-1, and others. In this respect, the increase in NK cell function induced by AMP-B may be attributed to an activation pathway unrelated to CD16.

The mechanisms underlying the AMP-B-induced increase in NK cell cytotoxicity involved increased conjugate formation and granule polarization. Although the increase in conjugate formation was maintained for a short time, it was sufficient to induce granule polarization, given an overall increase in NK cytotoxicity by AMP-B. This implies that a longer duration of cell–cell adhesion may not be necessary for effective cytotoxicity, which merits further investigation. Calcium flux and the expression of perforin and granzyme in NK cells were independent of the effect of AMP-B. AMP-B is suggested to be a TLR2 and TLR4 agonist [25]. The TLR4-stimulatory activity of AMP-B is similar to that of monophosphoryl lipid A, suggesting the presence of a Toll/IL-1R domain-containing adaptor inducing IFN-β (TRIF)-biased signaling. Although TLR2 and TLR4 are expressed on NK cells [26,27], it is unlikely that a TRIF-dependent pathway is directly responsible for the increase in NK cell activity induced by AMP-B [28]. Nevertheless, we do not exclude the possible involvement of other receptors or binding proteins in the stimulation of NK cells by AMP-B. If any of these are involved, they could be related to cell–cell interaction, lytic synapse formation, or granule polarization. In conclusion, repositioning of AMP-B as an immunomodulatory drug is a potential therapeutic strategy to improve immunotherapy, particularly for patients at potential risk of microbial infection, by increasing NK cell activity.

## 4. Materials and Methods

### 4.1. Cells and Reagents

Human blood samples from healthy donors were drawn for research purposes using a protocol approved by Asan Medical Center Institutional Review Board with informed consent. PBMCs were isolated using lymphocyte separation medium (MP Biomedicals, Santa Ana, CA, USA). The human NK cell line NKL (a gift of Michael J. Robertson) was cultured in RPMI 1640 supplemented with 10% fetal bovine serum (FBS), 1 mM sodium pyruvate, and 200 U/mL recombinant IL-2 (rIL-2) (Roche, Basel, Switzerland). K562 (ATCC CCL-243) and 221 (a gift of Jenny Gumperz and Peter Parham) cells were cultured in Iscove’s modified Dulbecco’s medium (IMDM) supplemented with 10% FBS and 2 mM l-glutamine. PBMCs and NK cells were activated with 200 U/mL rIL-2 for 24 h. The K562-mb15-41BBL cell line (a gift of Dario Campana) for NK cell expansion was cultured in RPMI1640 supplemented with 10% FBS. All chemicals were from Sigma unless indicated otherwise.

The following fluorochrome-conjugated antibodies were used in the flow cytometric analyses: anti-CD3-PerCP (SK7), anti-CD56-PE (NCAM16.2), and anti-CD107a-FITC (H4A3) (BD Biosciences, San Jose, CA, USA). For confocal microscopic analysis, CFSE, CellTracker orange CMTMR and Alexa Fluor 488-phalloidin were obtained from Invitrogen (Waltham, MA, USA), and Alexa Fluor 647-anti-perforin (dG9) was from Biolegend (San Diego, CA, USA).

### 4.2. NK Cell Expansion

Primary human NK cells were expanded as previously described [29] with some modifications. PBMCs (1.5 × 10^6^) were incubated in a 24-well tissue culture plate with 100 Gy-irradiated K562-mb15-41BBL cells (1 × 10^6^) in Stem Cell Growth Medium (SCGM; CellGenix, Freiburg, German) supplemented with 10% FBS and 10 U/mL rIL-2. The medium was exchanged every 2 days with fresh medium with rIL-2. After 1 week, residual T cells were depleted with a CD3 positive selection kit (STEMCELL Technologies, Cambridge, MA, USA). Purified NK cells were incubated in SCGM supplemented with 10% FBS, 100 U/mL rIL-2, and 5 ng/mL rIL-15 for 2 additional weeks with a medium exchange every 2 days. The expanded cell populations were 96–99% CD3−CD56+ as assessed by flow cytometry.

### 4.3. Compound Screening

Compounds that enhance NK cell cytotoxicity were screened from Prestwick Chemical Library (Prestwick-1200™, Prestwick Chemical, San Diego, CA, USA), which comprises 1,200 marketed drugs, using the Europium-based cytotoxicity assay [10]. An automated liquid handler (Perkin Elmer model AJM8M01, Perkin Elmer, Waltham, MA, USA) was used to dispense 20 µL of a single active compound (50 µM) into the wells of columns 2–11 of 96-well V-bottom plates (total, 15 assay plates). Next, 80 µL of NKL cells (2.5 × 10^4^) in IMDM medium was dispensed into wells containing 20 µL of 50 µM compound solutions. The cells were then incubated with compounds for 1 h at 37 °C. Then, 100 µL of BATDA (Perkin Elmer)-labeled 221 cells (5 × 10^3^) in Iscove's Modified Dulbecco’s Media (IMDM) medium containing sulfinpyrazone was added, mixed briefly, centrifuged at 30× *g* for 3 min and incubated for 2 h at 37 °C to achieve an effector:target (E:T) ratio of 5:1 and final assay concentrations of 5 µM per compound. After the incubation, the cells were pelleted by centrifugation, and the supernatant (20 μL) was assayed for Europium release to determine the effect of the examined compound on NK cell cytotoxicity. The percentage of specific cytotoxicity was calculated as [experimental release (counts) − spontaneous release (counts)]/[maximum release (counts) − spontaneous release (counts)] × 100. Spontaneous release was determined by incubating the targets in the absence of effector cells, and maximum release was determined by incubating the targets with 0.5% Triton-X. Wells in columns 1 and 12 were used to determine spontaneous release, maximum release, and experimental release for vehicle only (no compound). Raw values were transferred to Excel software to evaluate relative NK cell cytotoxicity.

### 4.4. Plate Configuration, Z′-Factor Calculation and Standard Score Normalization

In the primary screen, wells in column 2-11 harboring target cells with NK cells received a single test compound from Prestwick-1200™ library. The wells in column 1 were seeded with target cells without NK cells. In final evaluation step, wells of E to H received a detergent (0.5% Triton-X) to let all the amount of loaded europium be released into the media, while wells of A to D were subjected to measurement directly. The raw values from wells of A to D represented spontaneous release, while wells of E to H were considered maximum release. The wells of A to D in column 12 only harbored culture media to represent background value. The wells of E to H in column 12 were plated by NK cells with targets cells to induce the same experimental condition with 80 test wells in column 2-11. The raw values from wells of E to H in column 12 represent control reaction of NK cell-induced cytotoxicity. The assay stability of each plate was evaluated by Z′-factor [30]. In our screen, we considered the control reaction (wells of E to H in column 12) as positive (+) control, and the spontaneous release (wells of A to D in column 1) as negative (−) control. Z′-factor of each plate was calculated as:

(1) $$Z^{\prime }=1-\left(3SD_{+}+3SD_{-}\right)/\left|Ave_{+}-Ave_{-}\right|$$

To identify compounds capable of enhancing NK cell cytotoxicity, a standard score (Z-score) for each tested compound was calculated using the following equation: σ = (raw value of well − mean of total tested wells in a plate)/(standard deviation of total tested wells). The single bioactive compound which shows standard score (σ) higher than 2.0 was considered as primary hit.

### 4.5. Cytotoxicity Assay

For the Europium-based cytotoxicity assay, K562 or 221 cells were loaded with 40 μM BATDA (Perkin Elmer) for 30 min at 37 °C. Cells were then washed in a medium with 1 mM sulfinpyrazone (Sigma, St. Louis, MO, USA), resuspended at 1 × 10^6^ cells/mL in the medium, and incubated for 30 min at room temperature (RT). Cells were washed and incubated with effector cells in the presence of sulfinpyrazone for 1 h (primary expanded NK cells) or 2 h (NKL cells) at 37 °C. Plates were mixed briefly and centrifuged at 30× *g* for 3 min. The supernatant (20 μL) was incubated with 200 μL of 20% Europium solution (Perkin Elmer) in 0.3 M acetic acid for 5 min and detected with a VICTOR X4 multi-label plate reader (Perkin Elmer).

### 4.6. Assay of NK Cell Degranulation

NK cell degranulation was determined by the cell surface expression of CD107a as previously described [8,31]. Briefly, IL-2-activated PBMCs (2 × 10^5^ cells) were mixed with an equal number of K562 cells and incubated for 2 h at 37 °C. The cell pellets were resuspended in flow cytometry buffer (phosphate-buffered saline (PBS) with 2% FBS) and stained with anti–CD3-PerCP, anti–CD56-PE, and anti-CD107a-FITC antibodies for 30 min in the dark at 4°C. To determine the degranulation of primary expanded NK cells, NK cells were incubated with K562 cells in the presence of anti-CD107a-FITC antibody. Lymphocytes were gated on FSC and SSC characteristics, and the CD107a expression on CD3-CD56+ NK cells was analyzed using a flow cytometer (FACScanto II, BD) and FlowJo software (ver 9.7.6, Treestar, Ashland, OR, USA).

### 4.7. Conjugation Assay

The conjugation assay was performed according to a previously described protocol [32]. NKL cells loaded with CFSE and 221 cells labeled with CellTracker orange CMTMR (Molecular Probes, Waltham, MA, USA) were separately chilled on ice and then mixed at an E: T ratio of 1:1. Cells were spun down at 30× *g* for 3 min and then incubated at 30 °C for the indicated times. Thereafter, cells were moved to ice, fixed in PBS containing 4% paraformaldehyde, and washed twice with FACS buffer. Conjugates (CFSE+CMTMR+) were detected by flow cytometry.

### 4.8. Granule Polarization Assay

Polarization of perforin to target cells was examined as described previously with slight modifications [33]. To facilitate NKL cell identification, 221 target cells were first stained with CellTracker orange CMTMR according to the manufacturer‘s instruction. The CMTMR-labeled 221 cells were mixed with unlabeled NKL cells at a 1:1 ratio in serum-free IMDM medium and incubated for 30 min at 37 °C to allow conjugate formation. The cell suspension was subsequently transferred to coverslips coated with Cell-Tak (Corning Inc., Corning, NY, USA) and incubated for an additional 15 min at 37 °C for attachment. Thereafter, cells were fixed in PBS containing 4% paraformaldehyde, washed twice with PBS, permeabilized in PBS containing 1% bovine serum albumin (BSA), 0.2% Triton-X 100, and 0.1% sodium citrate, and blocked for 30 min at RT in PBS supplemented with 1% BSA and 1% goat serum. After washing, cells were stained for 90 min at RT with Alexa Fluor 488-phalloidin (Invitrogen) and Alexa Fluor 647-anti-perforin (Biolegend). After an additional wash, coverslips were mounted over glass slides using ProLong Gold antifade reagent (Molecular Probes, Waltham, MA, USA). Data were acquired using a laser-scanning microscope LSM 710 (Carl Zeiss, Oberkochen, Germany). Only conjugates where one NKL cell was conjugated with single 221 cells were analyzed. At least 100 different conjugates were analyzed for each condition. Conjugate stages were defined as follows: 0, conjugates lacking actin polymerization and granule polarization; 1, conjugates with polymerized actin without granule polarization; 2, conjugates in which perforin-containing granules partially polarized toward the lytic synapse; and 3, conjugates in which granules fully polarized toward the synapse.

### 4.9. Perforin and Granzyme B Staining of NK Cells

NKL cells (2 × 10^5^ cells) were incubated with different concentrations of Amp-B (1 or 5 µM) for 3 h at 37 °C, followed by incubation in BD Cytofix/Cytoperm solution. Before and after intracellular staining with anti–perforin-Alexa647 and anti-granzyme B-Alexa 647, the cells were washed twice with BD Perm/Wash buffer and then analyzed by flow cytometry.

### 4.10. Statistical Analysis

All the experiments were independently repeated at least two times. Statistical analyses of cytotoxicity and degranulation after treatment with different doses of Amp-B were performed by one-way ANOVA. Dunnett’s tests were performed for multiple comparison post-tests. For statistical analysis of conjugate formation, data for different groups were compared by two-way ANOVA. All statistical analyses were performed using GraphPad Prism 5.0. software (GraphPad Software, Inc., San Diego, CA, USA), and *p* Values < 0.05 were considered statistically significant.

## Abbreviations

NK	Natural killer
AMP-B	Amphotericin B
IMiD	Immunomodulatory drug
IFN	Interferon
IL	Interleukin
DC	Dendritic cell
MIP	Macrophage inducible protein
HTS	High throughput screening
PBMC	Peripheral blood mononuclear cell
LAK	Lymphokine-activated killer
ADCC	Antibody-dependent cellular cytotoxicity
IMDM	Iscove’s modified Dulbecco’s medium
